# Supplementary material for: “I think there is a certain worm that disturbs the head”: Community perspectives and care pathways for psychosis in Malawi
Source: PLOS Ment Health. 2026 May 7;3(5):e0000604. doi: 10.1371/journal.pmen.0000604 (PMC13152176; doi:10.1371/journal.pmen.0000604)
Supplement: Attachment — Submitted filename: Response to reviewers-PMED-D-25-00520.pdf [file pmen.0000604.s004.pdf]

No.	Reviewer comments	Author's response
	**Reviewer 1**	
1	**Topic**	
	Topic: Your topic, ""I think there is a certain worm that disturbs the head": Perspectives from traditional healers, religious leaders, caregivers, and people with lived experience of psychosis in Malawi" is not precise. In its present state, it looks a "sweeping statement", trying to generalize or include everything. Think critically and make a more precise title.	The title reflects a representative quote from the data, and helps to ensure the voices of respondents are reflected in the title. We have modified the title slightly to improve preciseness. The new title is:<br><br>*"I think there is a certain worm that disturbs the head"*: **Community perspectives and care pathways for psychosis in Malawi"**
2	**Introduction**	
	Again, the introduction is less focus because of the topic. The most confusing thing emanating from the topic is "Perspectives from traditional healers, religious leaders, caregivers, and people with lived experience of psychosis in Malawi". Perspective on what??? Just perspective makes it lose and less focus, thus affecting everything in your study, including result section.<br>The concluding part of your introduction, showing the research questions is very good. Use this to guide the structuring of your result section.	From line 54-64, we have added few sentences to elucidate what the perspectives entailed on relevance of included sub-sections of the introduction.
3	**Results**	
	First present a table summarizing the themes and sub-themes before you start using them in the findings. Do not remove table 1 but add another table of themes and sub-themes below it.<br>Remove all discussions from result section. In result section, present only what you found in relation to your study objectives.<br>The concluding part of your introduction section, showing the research questions is very good. Use this to guide the structuring of your result section. In its present form, the structure of the result section is not tailored to the	Thank you for these useful suggestions. A table of themes Table 2, lines and sub-themes plus representative quotes has been added to illustrate the structure of the results sections. We do not agree with the critique that the results section currently includes discission. We have re-organized the results section (and the new table) to correlate to the research questions. As noted, we have also removed some sentences from the Results section that were better suited to the Discussion section; these

	research questions or study objectives, making it difficult and boring to read.	are indicated in the tracked changes (for example, lines 327–328 and 393–395). Finally, we have added at least one representative quote in each subsection to further strengthen the presented themes and sub-themes.
4	**Discussion**	
	The discussion is also not clear because of the way results are presented. Once you have addressed the concerns in the result section, you can also aggressively revise the discussion section	The reviewer comments have not specifically indicated what aspects of the discussion should be revised. In keeping with an overall revision of the paper, we have edited the discussion to ensure clarity and conciseness.
	**Reviewer 2**	
1	Clarity on Ethical Approval Timeline<br>The cover letter explains the confusion regarding ethics approval dates, but the manuscript itself should briefly clarify in the Methods or Ethics Statement that approval was granted prior to data collection (July 2023 for October–December 2023 enrollment).	A statement has been added in the ethical approval section that ethics approval was granted prior to data collection-line 178-179
2	Data Saturation Justification<br>The authors reference Hennink et al. to justify sample size but could briefly elaborate on how saturation was assessed in practice (e.g., no new themes emerged in later interviews/FGDs).	A clarifying sentence has been added on how saturation was determined in this study "Based on this principle, after sixteen IDIs and four FGDs, no new themes emerged highlighting data saturation" -line 155-156
3	Generalizability and Context<br>The Limitations section appropriately notes that findings may not be generalizable to all of Malawi. Consider briefly discussing how findings might resonate with (or differ from) other rural, low-resource settings in sub-Saharan Africa.	A sentence has been added to highlight resonance with findings from similar settings " despite consistency with findings from similar low-resource settings in sub-Saharan Africa [6,7]" line 505-507
4	**Minor Writing Edits:**<br>**Check for minor grammatical issues (e.g., "No any competing interests" → "The authors declare no competing interests").**<br>**In conclusion. Cultural norms… → should be one sentence, not split.**<br>**Ensure consistency in abbreviations (e.g., PWLE is defined but sometimes**	Thank you for your meticulous grammar check. This has been corrected: "PWLEs" has been revised to "PWLE." The conclusion sentence has been combined. Lastly, per your suggestion of consistency, we have opted to use cannabis throughout the manuscript.

	**written as PWLEs (e.g., 146 line number)) and “Indian hemp” vs “cannabis” (both are fine, but consistency matters**	
