# Supplementary material for: “I think there is a certain worm that disturbs the head”: Community perspectives and care pathways for psychosis in Malawi
Source: PLOS Ment Health. 2026 May 7;3(5):e0000604. doi: 10.1371/journal.pmen.0000604 (PMC13152176; doi:10.1371/journal.pmen.0000604)
Supplement: Attachment — Submitted filename: PLOS Mental Health Cover Letter_response to reviewers.docx [file pmen.0000604.s003.docx]

Dennis Chasweka

Department of Bioethics and Behavioural Social Sciences

Kamuzu University of Health Sciences

P/Bag 360

Blantyre, Malawi

19^th^ November, 2025

The Editorial Team

PLOS Mental Health

Dear Editors,

**RE: Re-Submission of Manuscript Titled *“I think there is a certain worm that disturbs the head”: Perspectives from traditional healers, religious leaders, caregivers, and people with lived experience of psychosis in Malawi.***

I write to respond to the editorial team on the recent query raised that the manuscript appears to be a resubmission of PMEN-D-25-00460, which was initially rejected. The reason provided by the editorial team (via Dr. Jayati Bhattacharyya on 6^th^ November) stated that “*Based on our evaluation of your manuscript and the information you provided, we do not feel that your submission meets our ethical requirements for human subjects research submissions.* I immediately inquired for clarity as this seems to be serious breach for any researcher. I received clarification from (Meghan hom) that “*Thank you for your query and interest in long-term ethical standards. An ethical requirement that PLOS has for authors is to receive ethical approval prior to study recruitment. If you feel that you have indeed met this requirement, you are welcome to resubmit your research”.* I responded that the study had ethical approval before any enrolment occurred and provided the ethical clearance dated 31^st^ July, 2023. Enrolment happened between October and December, 2023. For this reason, I am justified I met the PLOS ethical requirement, and hence the resubmission of the manuscript. The ethical clearance has been updated in the attachment files. Below is the email trail of all communication with PLOS editorial team prior to submission.

Please, do not hesitate to contact me if there is still need for more clarity. Otherwise, I hope this will straighten things up and review process could proceed.

Kind regards,

Dennis Chasweka(Lead Author)

**Email 1: Editorial decision to reject the manuscript**

From: **PLOS Mental Health** <[em@editorialmanager.com](mailto:em@editorialmanager.com)>
Date: Thu, 6 Nov 2025, 6:07 am
Subject: PLOS Mental Health Decision: PMEN-D-25-00460 - [EMID:20218fc617a5433b]
To: Dennis Chasweka <[dchasweka@medcol.mw](mailto:dchasweka@medcol.mw)>

CC: [jayati@newgen.co](mailto:jayati@newgen.co)

PMEN-D-25-00460

“I think there is a certain worm that disturbs the head”: Perspectives from traditional healers, religious leaders, caregivers, and people with lived experience of psychosis in Malawi.

PLOS Mental Health

Dear Dr. Chasweka,

Thank you very much for submitting your manuscript for review by PLOS Mental Health. After careful consideration, we have decided that your manuscript does not meet our criteria and must be rejected.

Based on our evaluation of your manuscript and the information you provided, we do not feel that your submission meets our ethical requirements for human subjects research submissions. PLOS Mental Health requires that research meets all applicable standards for the ethics of experimentation and research integrity ([http://journals.plos.org/plosone/s/human-subjects-research](http://track.editorialmanager.com/CL0/http:%2F%2Fjournals.plos.org%2Fplosone%2Fs%2Fhuman-subjects-research/1/010f019a57593ef1-8d9bf05f-8764-492b-85de-875b1130113f-000000/WP-SOtYmlB4DGUuKnwZNcx5LCKX8iDETbhfuGDzWPbI=236)). We reserve the right to reject any submission that does not meet our internal ethical standards, which in some cases are more stringent than local ethical standards.

As a result of these concerns we cannot consider the manuscript for publication at PLOS Mental Health. I am sorry that we cannot be more positive on this occasion, but hope that you understand the reasons for this decision.

Yours sincerely,

Jayati Bhattacharyya, PhD
Editorial Office
PLOS Mental Health

Additional Editor Comments (if provided):

**Email 2: My Request for clarity on rejection decision**

**From:** Dennis Chasweka [[dchasweka@medcol.mw](mailto:dchasweka@medcol.mw)]
**Sent:** 11/6/2025, 2:45 PM
**To:** [mentalhealth@plos.org](mailto:mentalhealth@plos.org)
**Cc:** [jayati@newgen.co](mailto:jayati@newgen.co)
**Subject:** Re: Your PDF “I think there is a certain worm that disturbs the head”: Perspectives from traditional healers, religious leaders, caregivers, and people with lived experience of psychosis in Malawi. has been built and requires approval - [EMID:451518748a8dccb4]

[CAUTION: External email. Proceed with Caution. ]

Dear Dr  Bhattacharyya  ,

Thanks so much for your feedback on the manuscript I submitted. I truly appreciate the time and effort you took to review the manuscript to arrive at the final decision of rejection. The Editor's decision is therefore respected. However, for future submission, I would just like to seek specific ethical requirements where the standards were not met. The feedback states "Based on our evaluation of your manuscript and the information you provided, we do not feel that your submission meets our ethical requirements for human subjects research submissions". A human subjects research form and two ethics committees approvals were submitted and it will really be significant as a mental health researcher to specify requirements or areas not fulfilled/missing that need to be considered for future submissions to PLos Mental Health. Thanks once again and looking forward to your response.

Kind regards,

Dennis

**Email 3: Response from editorial team on my request for clarity**

--------------- Original Message ---------------
**From:** PLOS Mental Health [[mentalhealth@plos.org](mailto:mentalhealth@plos.org)]
**Sent:** 11/7/2025, 12:10 AM
**To:** [dchasweka@medcol.mw](mailto:dchasweka@medcol.mw)
**Subject:** Re: Your PDF “I think there is a certain worm that disturbs the head”: Perspectives from traditional healers, religious leaders, caregivers, and people with lived experience of psychosis in Malawi. has been built and requires approval - [EMID:451518748a8dccb4]

Dear Dr. Chasweka, 
 
Thank you for your query. 
 
Your email has been forwarded to the relevant team, who will be in touch with you as soon as possible. 
 
If you have any questions in the meantime, please don’t hesitate to ask. 
 
Kind regards, 
 
Kate Anne Dimayuga 
PLOS Mental Health 

Case Number: 09325348

**Email 4: Editorial response to my query on Clarity and rejection Decision**

On Thu, Nov 13, 2025 at 3:26 AM PLOS Mental Health <[mentalhealth@plos.org](mailto:mentalhealth@plos.org)> wrote:

Dear Dr. Chasweka,

Thank you for your query and interest in long-term ethical standards. An ethical requirement that PLOS has for authors is to receive ethical approval prior to study recruitment. If you feel that you have indeed met this requirement, you are welcome to resubmit your research.

Kind regards,
Meghan Hom
Peer Review Operations Specialist

PLOS | [plos.org](http://plos.org/)
Empowering researchers to transform science
1265 Battery Street, Suite 200, San Francisco, CA 94111
[plosone@plos.org](mailto:plosone@plos.org)

Case Number: 09325348
thread::Cwd6UQRwdMCSxXIREShbMRo::

**Email 5: My response to editorial team on Justification to resubmit**

From: **Dennis Chasweka** <[dchasweka@medcol.mw](mailto:dchasweka@medcol.mw)>
Date: Thu, 13 Nov 2025, 11:00 am
Subject: Re: Your PDF “I think there is a certain worm that disturbs the head”: Perspectives from traditional healers, religious leaders, caregivers, and people with lived experience of psychosis in Malawi. has been built and requires approval - [EMID:451518748a8dccb4]
To: PLOS Mental Health <[mentalhealth@plos.org](mailto:mentalhealth@plos.org)>

Dear Megham and the Editorial Team,

Thank you for your feedback. I would like to confirm that ethical approval for this study was obtained prior to participant recruitment. The approval was granted on **31 July 2023**, and study enrolment took place between **October and December 2023** (please see the attached Malawi ethics approval).

It appears that the ethics approval certificates included in the previous submission were for the next phase of the study, which was approved more recently—hence the confusion.

I would therefore like to proceed with resubmission for your consideration. Thank you for your understanding.

Kind Regards,

Dennis

**Attachment for ethical approval updated:**

The ethical clearance in question has been attached in Attach files (other) is named **: Lawrie amended approval_July 2023.pdf**
